# Fatigue Life Estimation with Mean Stress Effect Compensation for Lightweight Structures—The Case of GLARE 2 Composite

**DOI:** 10.3390/polym12020251

**Published:** 2020-01-21

**Authors:** Michał Böhm, Karolina Głowacka

**Affiliations:** Faculty of Mechanical Engineering, Opole University of Technology, Ul. Mikołajczyka 5, 45-271 Opole, Poland; k.glowacka@po.edu.pl

**Keywords:** mean stress, fatigue life prediction, lightweight structures, Glare II composite

## Abstract

This paper describes the current state-of-the-art in fatigue life assessment for lightweight composite structures with the use of the frequency domain fatigue life calculation method. Random stationary gaussian loading signals have been generated and served in the process of fatigue calculation. The material information that is being used in the calculation process has been obtained from literature for the Glare 2 composite. The effect of nonzero mean stress and different fiber orientations have been taken into account. The calculations have been performed for two mean stress compensation models by Goodman and Gerber. The proposed procedure gives satisfying results for the high-cycle fatigue region for Goodman and an overall good comparison in both regimes for the Gerber model.

## 1. Introduction

The purpose of the work was to analyze the effect of the nonzero mean stress value on the fatigue lifetime estimation method defined in the frequency domain in hybrid materials. The analysis of the mean stress effect plays an important role when estimating the fatigue life of materials due to the fact that it can be considered as an extra static load added to the system. This can be also analyzed as an effect of prestressing the composite material, which is analyzed with the crack propagation approach by many scientists like Park et al. [1] or Guo et al. [2]. However, up to now, it was usually considered for isotropic materials, and rarely was discussed in terms of durability up to the crack initiation stage. There is a noticeable increase in interest in the frequency domain fatigue life estimation methods, as can be noticed by the number of publications related to this area published in the recent years by the research groups of Benasciutti et al. [3,4], Cianetti et al. [5,6], and Slavic et al. [7,8].

In the present paper the mean stress effect compensation was analyzed for materials consisting of three layers of aluminum and two layers of Glass Fiber Reinforced Polymer (GFRP). For this purpose, two different models were used for three different fiber orientations—θ = 0°, θ = 5°, and θ = 10°. Another important aspect was the comparison between rainflow and frequency domain methods for the high and low-cycle fatigue regime. Fatigue lifetime prediction is a constantly developing area for Carbon Fiber Reinforced Polymer (CFRP) materials as we can notice by the number of publications [9].

Issues related to the determination of fatigue life of materials have been analyzed for many years. The fact of material cracking due to cyclic loads with an amplitude much smaller than the maximum static stress value was observed for the first time in the 19th century [10].

At the beginning, fatigue was mainly analyzed in isotropic materials, such as steel. However, with the passage of time and the development of materials, fatigue was also analyzed in modern materials, including those of an anisotropic nature. Nowadays when designing elements, a lot of emphasis is put on lightweight structures, such as those made of composite laminates or fiber-metal laminates, for which classic methods used for e.g., steel cannot be used.

The fatigue phenomenon in lightweight structures is a complex matter and many factors are causing it. We can divide these factors into material microscopic structure as well as loading conditions and working forces. In terms of this research, we are going to discuss the problem of static forces acting on the whole structure. This is the case of nonzero mean stresses that are acting on the structures. The own weight of the construction as well as a static force acting on the lightweight structure may influence its fatigue life enormously. During the fatigue life assessment process, it is crucial to take into account cases of nonzero mean stresses. We have to compensate this effect in some kind of way. The main solution that is used is based on the idea of amplitude transformation in terms of mean stress. This idea works well for the case of algorithms defined in the time domain, but in the case of frequency domain algorithms we need to use a different path. This path includes a transformation process of the power spectral density (PSD) and works well for metallic materials. There are no papers analyzing this method for composite materials with the effect of nonzero mean stress. The aim of this paper is to perform a simulation of fatigue life assessment for a randomly generated signal and material constants taken out of the literature for composite materials under nonzero mean stresses. 

In order to understand the idea behind amplitude transformation, we need to define some basic definitions used in the process.

Stress amplitude can be defined as (Equation (1)):

(1) $$\sigma_{a}=\frac{\sigma_{max}-\sigma_{min}}{2}\;$$

where *σ_max_* and *σ_min_* are the maximum and minimum stress.

The mean stress can be defined as (Equation (2)):

(2) $$\sigma_{m}=\frac{\sigma_{max}+\sigma_{min}}{2}\;$$

**Figure 1:**
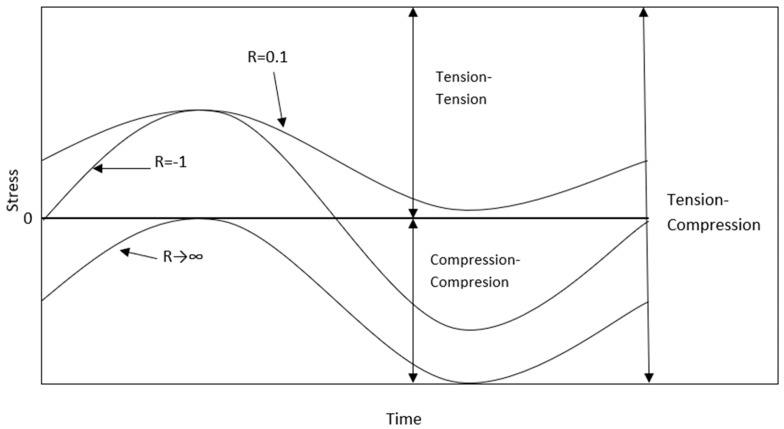
Different stress ratio cases.

Another important feature related to loading conditions is the stress ratio R. Some interesting cases of this ratio are presented in Figure 1. The R ratio can be defined as (Equation (3)):

(3) $$R=\frac{\sigma_{min}}{\sigma_{max}}\;$$

**Figure 2:**
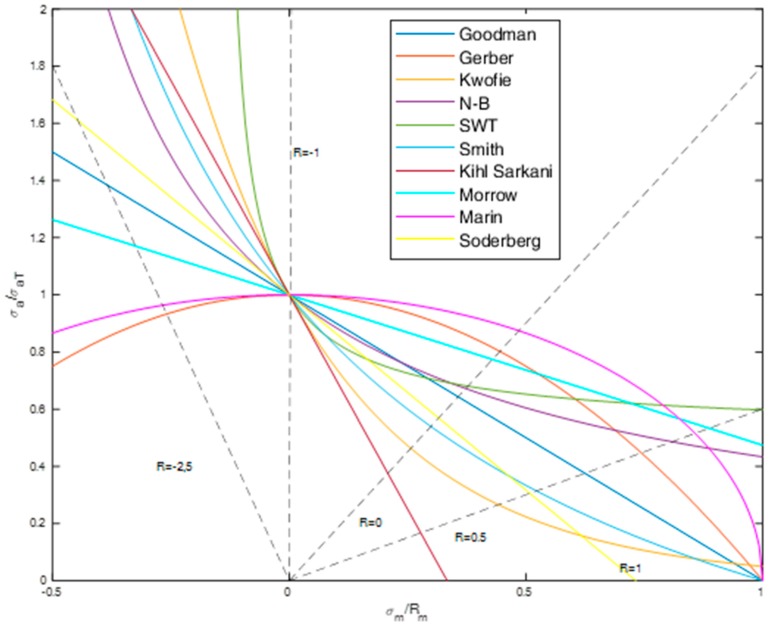
Some of the most popular mean stress compensation models.

The nonzero mean stress has a huge effect on the fatigue life and is often taken into account in the transformation process of stress amplitudes. These transformations can be presented in the form of normalized graphs in terms of mean stress to ultimate strength and stress amplitude to transformed amplitude with many literature models, as presented in Figure 2. The graph is describing the safe area and the dangerous areas in relation to fatigue life. The safe area is always below the chosen mean stress compensation model. These areas can be also divided into the tension and compression site, which is divided by the vertical line R = –1. These types of graphs are often used to define the material sensitivity to mean stress. The most popular is the Goodman [11] model, which serves as one of the oldest mean stress compensation models (Equation (4)):

(4) $$\frac{\sigma_{a}}{\sigma_{aT}}=1-\frac{\sigma_{m}}{R_{m}}$$

where *σ_aT_* is the transformed stress amplitude and Rm is the ultimate tensile strength.

This model is linear, but we can also use a parabolic model proposed by Gerber [12], which can be defined as (Equation (5)):

(5) $$\frac{\sigma_{a}}{\sigma_{aT}}=1-{\left(\frac{\sigma_{m}}{R_{m}}\right)}^{2}$$

## 2. Materials and Methods 

### 2.1. Material GLARE 2

In this study, results of fatigue lifetime for different types of the GLARE 2 materials were analyzed. The results of experimental tests have been taken from the paper by Kawai and Kato [13]. In general, this material is a type of fiber-metal laminate (FML), which means that it is a combination of layers made of fiber-reinforced composite and layers made of metal. Such hybrid materials are widely used for the aircraft industry because of their relatively good mechanical properties in comparison with fiber-reinforced composites without metal layers. FMLs also offer lower mass in comparison with metal elements [14].

It can be assumed that FML materials use the best features of both composite and metal materials. It is assumed that individual components of FML materials eliminate each other′s disadvantages. The use of FMLs results in e.g., better fatigue life or improvement of fire-retardant properties. The external metal layers in FMLs protect the internal part of the laminate by compensating mechanical impact and also hydrothermal influence [15], meanwhile the use of composite materials protects elements from corrosion. 

**Figure 3:**
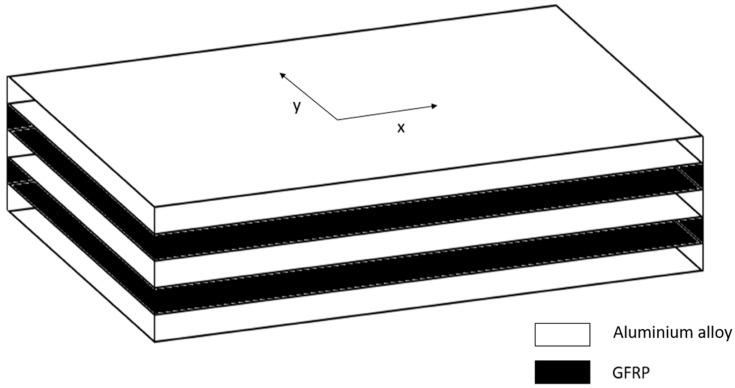
Scheme of the structure of specimens for which the fatigue lifetime was analyzed.

In this special case, the material consists of alternating layers of epoxy reinforced with unidirectional fibers and layers of aluminum alloy, as shown in Figure 3. There were 3 layers made of aluminum alloy—each layer had thickness of 0.211 mm, and 2 layers of GFRP—each layer had a thickness of 0.264 mm. As a result, specimens whose fatigue lifetime results were analyzed to assess the mean value had dimensions l = 200 mm, w = 25 mm, and h = 1.16 mm. The effect of fiber configuration on the tensile behavior of similar material configurations was presented and widely discussed in the recent paper by Bazli et al. [16].

In the literature [13] that was used in the process of data acquisition for calculations, it may be found that the basic static strength parameters of such material are:

- Strength in the axis parallel to the fibers X = 1144 MPa;
- Strength in the axis perpendicular to the fibers Y = 261 MPa.

**Figure 4:**
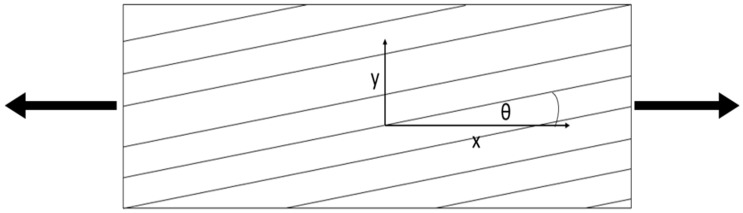
Scheme of the structure of specimens for which the fatigue lifetime was analyzed with axial loading directions.

As was introduced, in GLARE 2 materials, two layers of material are made of GFRP in which the epoxy resin is used as a matrix, while the continuous glass fiber is applied as reinforcement. However, the direction of fiber arrangement in the layer relative to the x direction marked in Figure 4 is not specified.

When the angle θ between fiber direction and the x direction is 0° ≤ θ ≤ 15°, the fatigue fracture has a type of fracture corresponding to the composite material. However, when 30° ≤ θ ≤ 90°, the fatigue fracture in the FML is characterized by the fracture of aluminum alloy. In this case, just the situation of the fatigue failure for the composite has been analyzed, which means that just the cases of θ ≡ 0°, θ ≡ 5°, and θ ≡ 10° have been analyzed. As a result, three different types of specimens have been taken into account:

- Al/GFRP θ ≡ 0°/Al/GFRP θ ≡ 0°/Al;
- Al/GFRP θ ≡ 5°/Al/GFRP θ ≡ 5°/Al;
- Al/GFRP θ ≡ 10°/Al/GFRP θ ≡ 10°/Al.

### 2.2. Fatigue of Lightweight Structures—The Special Case of Composite Materials

When analyzing the fatigue lifetime for composite materials there are three different models that can used [17]:

- Based on the S–N curves and failure criteria;
- Residual strength models;
- Progressive fatigue damage models.

One of the proposals of predicting the fatigue life was proposed by Zhou and Wu [18]. They adopted the fatigue master curves to obtain S–N curves of unidirectionally fiber-reinforced polymers. Then they determined the fatigue failure by Puck’s criterion. They obtained comparable results from the model they proposed and the experimental data.

In the literature, there may also be found tests where the influence of the temperature on the fatigue life was tested. Song et al. [19] tested fatigue life and the failure mechanism in carbon-reinforced 2.5 D woven composites at different temperatures (20° and 180°). As can be noted, the fatigue lifetime of composite materials has been lately widely analyzed.

The fatigue life in the special form of lightweight structures—fiber-metal laminates—was also separately analyzed. In FMLs, just as in other hybrid materials, the phenomenon of fatigue crack initiation is said to be associated with a metal component, and crack initiation occurs in the same conditions as the initiation in solid metal material [20]. Next, the fatigue crack propagation has to be considered in another way, as the composite part has a big impact on the behavior. After the occurrence of cracks in the metal layer, the fiber layers remain intact and transfer part of the energy derived from the crack. It leads to approximately linear growth of the crack propagation. Fatigue cracks in FML materials are divided into two groups:

- Running only in the metal part;
- Resulting delamination between the metal and the fibrous layer.

Therefore, they were analyzed as separate fracture mechanisms. The type of fracture mechanism depends on the rate of release of the deformation energy, which is also influenced by the interface geometry and the percentage of fibers in the composite layer. 

The fatigue process for composite materials may also be divided into two parts—the first for the crack initiation and the second for the crack propagation. The methods used in this paper refer to the first part where crack initiation is the final frontier for fatigue.

When the angle θ between fiber direction and the x direction is 0° ≤ θ ≤ 15°, the fatigue fracture has a type of fracture corresponding to the composite material. However, when 30° ≤ θ ≤ 90°, the fatigue fracture in the FMLs is characterized by the fracture of aluminum alloy. In this case, just a situation of the fatigue failure as for the composite.

### 2.3. Frequency Domain Calculation Methods

Fatigue life assessment techniques are set either in the time or frequency domain. Those set in the time domain are called the cycle counting methods and use algorithms for cycle counting. The most famous method, developed by Endo [21] and widely presented in the paper by Murakami [22], is called the rainflow cycle counting technique. Frequency domain methods are called spectral methods and use the power spectral density (PSD) to calculate the spectral moments, which are used in later steps to calculate the probability density function (pdf) to finally calculate fatigue life. The PSD can be calculated with the Equation (6):

(6) $$G_{x}\left(f\right)=2\int_{-\infty }^{\infty }R_{x}\left(\tau \right)e^{-j2\pi f\tau }d\tau \;$$

where *R*_x_ is the autocorrelation function.

To calculate the spectral moments we can use the Equation (7)

(7) $$\xi_{k}=\int_{0}^{\infty }G_{\sigma }\left(f\right)f^{k}df\;$$

We need to remember that in the case of nonzero mean stresses we need to perform a PSD transformation; in many terms, a correction to the PSD with the use of the Equation (8) proposed by Niesłony and Böhm [23]:

(8) $$G_{\sigma T}\left(f\right)=K^{2}\left(\sigma_{m}\right)G_{\sigma }\left(f\right)$$

where we can use classic formulas for mean stress compensation, such as the one proposed by Goodman, written in the form (Equation (9)):

(9) $$K_{Go}=\frac{1}{1-\frac{\sigma_{m}}{R_{m}}}$$

Or the one proposed by Gerber [12] (Equation (10)):

(10) $$K_{Go}=\frac{1}{1-{\left(\frac{\sigma_{m}}{R_{m}}\right)}^{2}}$$

After the transformation process, we can use the spectral moments to calculate the probability density function, i.e., with the Dirlik [24] model:

(11) $$p\left(\sigma_{a}\right)=\frac{1}{2\sqrt{\xi_{0}}}\cdot \left[\frac{K_{1}}{K_{4}}\cdot e^{\frac{-Z}{K_{4}}}+\frac{K_{2}\cdot Z}{R^{2}}\cdot e^{\frac{-Z^{2}}{2\cdot R^{2}}}+K_{3}\cdot Ze^{\frac{-Z^{2}}{2}}\right]$$

where Z, K1, K2, K3, K4, R—factors which are functions of the first five moments of the transformed PSD.

Afterwards we can use this information in order to calculate the fatigue life (Equation (12)):

(12) $$T_{cal}=\frac{1}{E\left[P\right]\int_{0}^{\infty }\frac{p\left(\sigma_{a}\right)}{N\left(\sigma_{a}\right)}d\sigma_{a}}\;$$

where the *N(σ_a_)* is the number of cycles from the Wöhler characteristic and *E[P]* is the expected number of peaks in the unit of time.

This method has been compared with the rainflow cycle counting method and the results have been presented in the next chapter.

## 3. Results

**Figure 5:**
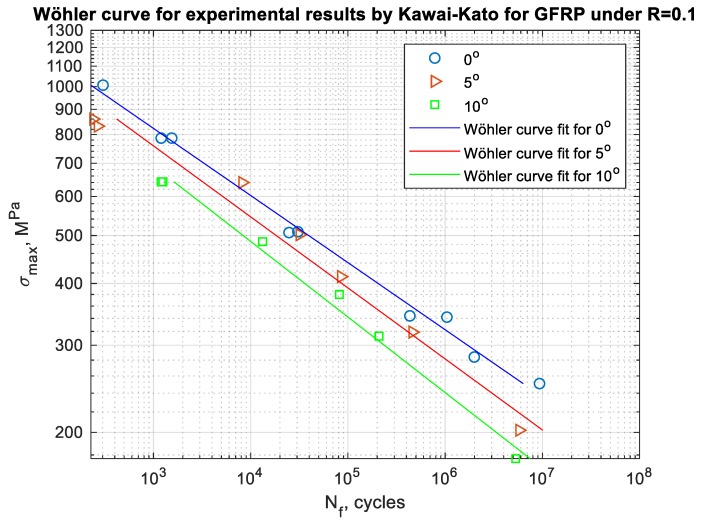
S–N curves for experimental results by Kawaii–Kato for GFRP for the fiber orientation 0, 5, and 10 degrees and R = 0.1.

One of the most important parts in the process of fatigue life estimation is the proper use of fatigue curve data, such as the crack propagation curves as presented by Guo et al. [25] or S–N curves which is the case in this paper [26]. The calculations have been performed for the composite material Glare 2 experiments performed by Kawai and Kato [13]. Three fiber orientations have been taken into consideration while analyzing the paper. Besides this, a crucial factor, which is the nonzero mean stress, has been also an object of interest. Composites tend to be used under the case of tension. Due to this, the stress ratio R = 0.1 that lies in the area of tension–tension has been taken for analysis. The S–N curves for fiber orientation under 0, 5, and 10 degrees and R = 0.1 used in the fatigue assessment process are presented in Figure 5. 

**Figure 6:**
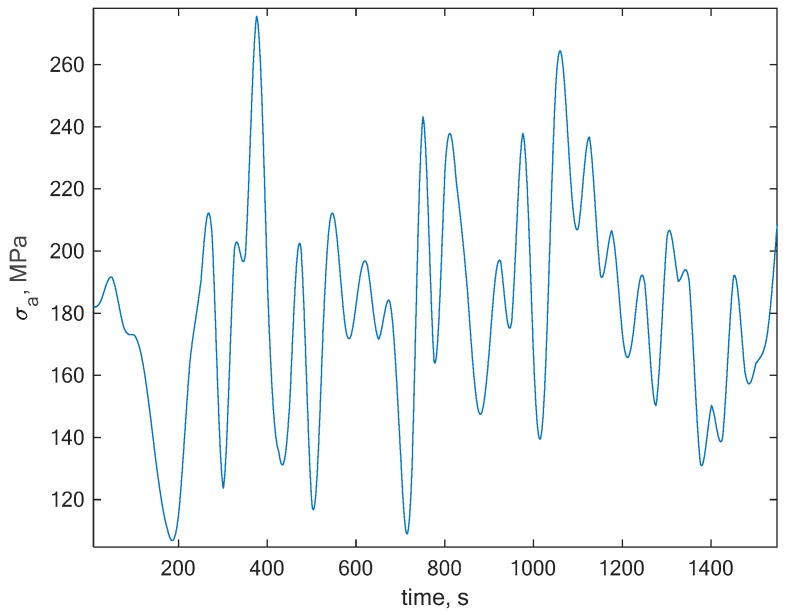
A section of the generated random nonzero mean stress characteristic with the global R = 0.1.

The next step was the random signal generation with nonzero mean stress. The signal had to have a gaussian and stationary characteristic in order to be used directly within the frequency domain. A random signal that suited these assumptions has been generated with the global value of R = 0.1 and a section of this signal is presented in Figure 6.

**Figure 7:**
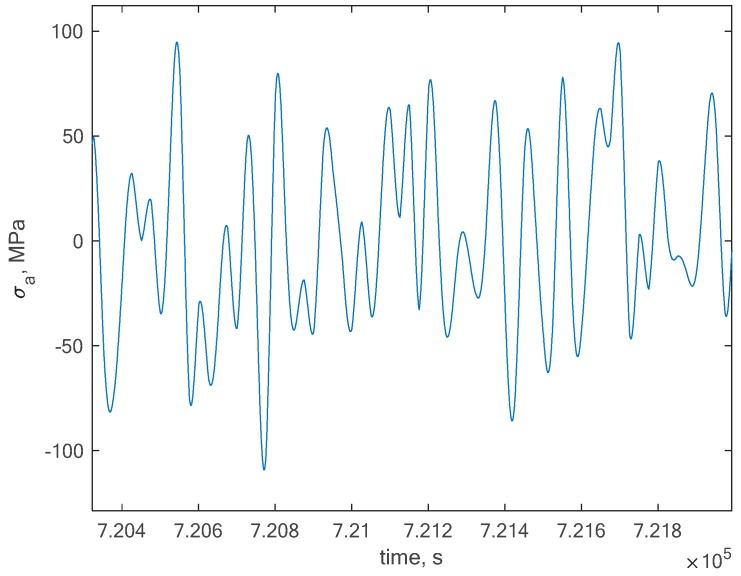
Transformed original stress signal in order to obtain a zero mean stress characteristic.

This signal has been directly taken for calculations for rescaled global maximum amplitudes with the use of the rainflow method. In order to calculate the fatigue life, we needed to transform the original signal in order to set a zero mean stress value, but we needed to remember the original mean stress in order to take it into account in the compensation process. In this case, we obtained a global zero mean stress history as presented in Figure 7.

**Figure 8:**
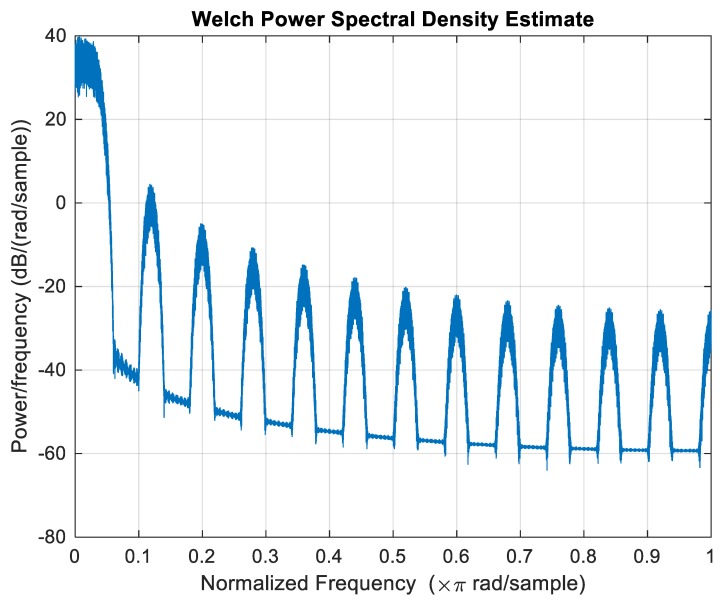
Power spectral density of the generated signal after zeroing the mean stress value.

The Welch PSD estimate of the generated random signal after zeroing the mean is presented in Figure 8.

**Figure 9:**
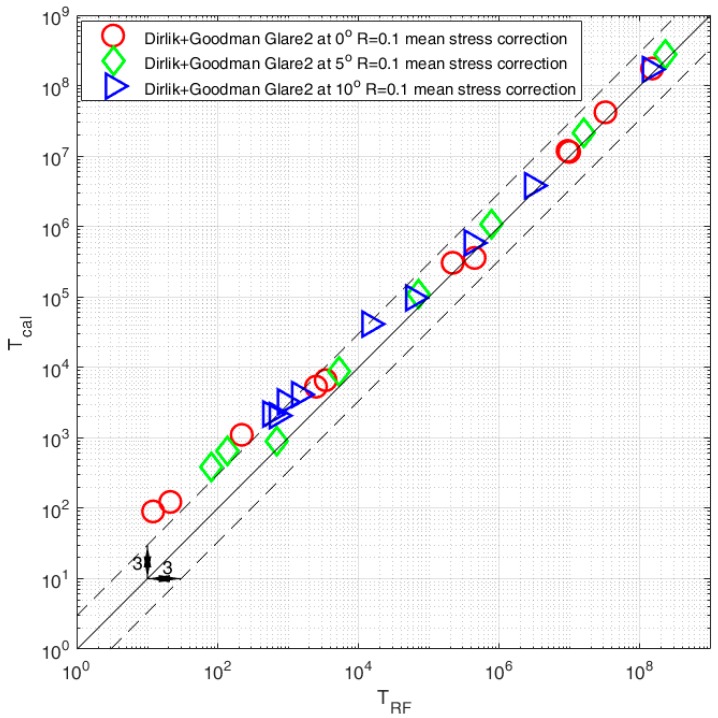
Comparison of fatigue life calculated with the use of the rainflow cycle counting method and the spectral method with the use of the Dirlik model with mean stress compensation with the Goodman model for three fiber orientations θ = 0°, θ = 5°, and θ = 10°.

**Figure 10:**
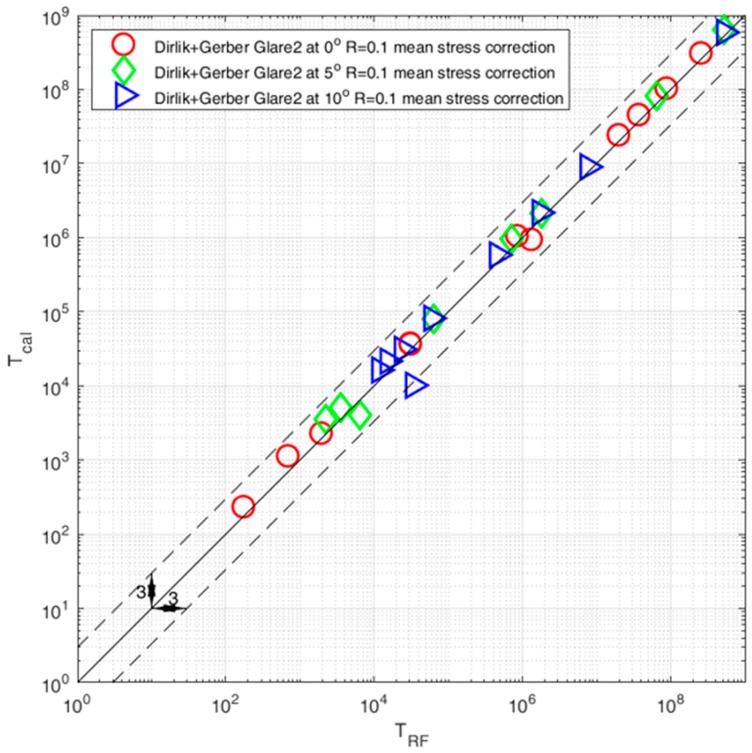
Comparison of fatigue life calculated with the use of the rainflow cycle counting method and the spectral method with the use of the Dirlik model with mean stress compensation with the Gerber model for three fiber orientations θ = 0°, θ = 5°, and θ = 10°.

The comparison of results obtained with the rainflow and the Dirlik model together with the Goodman and Gerber [11,12] mean stress compensation are presented in Figure 9 and Figure 10. The results are presented for three different fiber orientations θ = 0°, θ = 5°, and θ = 10°. The calculation time for the rainflow cycle counting method was between 40 to 60 s, in comparison to the frequency calculations took between 9 to 14 s.

## 4. Conclusions

As we can see, the topic of fatigue lifetime prediction is complex and one of the important factors is definitely the proper mean stress compensation approach. The calculation results will serve the authors in the process of fatigue experiment tests for their own fiber composition for different orientations and prestressed with a similar stress ratio of R = 0.1. The obtained results allowed the authors to formulate some conclusions:

- Composites require mean stress compensation in the fatigue life assessment process;
- Comparison between the rainflow and frequency domain method shows good combability in the high-cycle fatigue regime N > 103—in this case most of the results are within the scatter area of three;
- Comparison in the area of low-cycle fatigue regime N < 103 shows that the frequency domain method together with the Goodman model overestimated fatigue results—in this case most results are outside the scatter area of three;
- Comparison with the use of the Gerber mean stress compensation model shows overall good compatibility between rainflow and frequency calculations. One thing that we can notice is that the calculations with the Gerber model gave results that were over the low-cycle fatigue area;
- The computation time for the frequency domain method was a fifth of the time in comparison with the rainflow cycle counting method.
